# Making core outcomes more clinically meaningful: 2-for-the-price-of-1 with a distributional approach

**DOI:** 10.1186/1745-6215-16-S1-P16

**Published:** 2015-05-29

**Authors:** Janet L Peacock, Jessica W Lo, Odile Sauzet

**Affiliations:** 1 Division of Health and Social Care Research, King’s College London, London, SE1 3QD, UK; 2 NIHR Biomedical Research Centre at Guy’s and St Thomas’ NHS Foundation Trust and King's College London, London SE1 3QD, UK; 3 AG Epidemiologie & International Public Health, Bielefeld University, Postfach 10 01 31 D-33501 Bielefeld, Germany

## Background

In RCTs with a continuous outcome, it is easier to interpret a difference in proportions at high risk than the difference in means. For example when comparing lung function in two groups, a difference in proportion <10^th^ centile may be more clinically meaningful than the difference in means. However choosing a dichotomised continuous outcome at the outset requires a larger sample than using the raw data and may also distort the true difference since data are discarded on dichotomisation.

## Materials and methods

We have derived a dual-outcome solution where the difference in means and difference in proportion at high risk are equivalent inference-wise [1]. The distributional approach treats the proportion below a cut-point as a function of the mean and standard deviation of the distribution, assuming the data are Normal or can be transformed to Normal. The method is robust in situations commonly encountered [2]. We illustrate here with lung function (LF) outcome data [3].

## Results

This trial compared LF in adolescent children. They had been randomised to one of two types of ventilation at birth. The primary LF outcome forced expiratory flow 75% z-score (FEF_75_) differed significantly between the groups (difference in z-scores 0.23; 95% CI: 0.02, 0.45, p=0.04). The difference in percentage <10^th^ centile-for-normal was calculated using the distributional approach as: 10.3%; 95% CI: 0.7, 20.0%, p=0.04.

## Conclusions

Using the distributional approach an outcome can be presented as both a difference in means and difference in proportions at high risk without penalty due to loss of power or multiple testing since the test of the means is equivalent to the test of the proportion at high risk.
